# Aberrant blood MALT1 and its relevance with multiple organic dysfunctions, T helper cells, inflammation, and mortality risk of sepsis patients

**DOI:** 10.1002/jcla.24331

**Published:** 2022-03-09

**Authors:** Yibin Wang, Qinghe Huang, Fuyun He

**Affiliations:** ^1^ Department of Central Intensive Care Unit Zhongshan Hospital Affiliated to Xiamen University Xiamen China

**Keywords:** mucosa‐associated lymphoid tissue lymphoma translocation protein 1, multiple organic dysfunctions, prognosis, sepsis, T helper cell

## Abstract

**Background:**

MALT1 is linked with multiple organic dysfunctions, inflammatory storm, and T helper (Th) cell differentiation. Herein, the current study aimed to investigate the correlation of peripheral blood mononuclear cell (PBMC) MALT1 with Th1 cells, Th17 cells, and prognosis of sepsis patients.

**Methods:**

In general, 78 sepsis patients and 40 health controls (HCs) were enrolled. MALT1 expression was detected in PBMCs from all subjects by RT‐qPCR. Besides, Th1 and Th17 cells were measured in PBMCs from sepsis patients by flow cytometry; interleukin 17A (IL‐17A) and interferon gamma (IFN‐γ) were determined in serum from sepsis patients by ELISA.

**Results:**

MALT1 expression was higher in sepsis patients than HCs (*p* < 0.001). MALT1 expression was positively correlated with Th17 cells (*r*_s_ = 0.291, *p* = 0.038) and IL‐17A (*r*_s_ = 0.383, *p* = 0.001), but not with Th1 cells (*r*_s_ = 0.204, *p* = 0.151) or IFN‐γ (*r*_s_ = 0.175, *p* = 0.125) in sepsis patients. MALT1 expression was positively correlated with APACHE II score (*r*_s_ = 0.275, *p* = 0.015), C‐reactive protein (CRP) (*r*_s_ = 0.257, *p* = 0.023), and sequential organ failure assessment (SOFA) score (*r*_s_ = 0.306, *p* = 0.006) (MALT1 expression was positively correlated with SOFA respiratory system score (*r*_s_ = 0.348, *p* = 0.002), and SOFA liver score (*r*_s_ = 0.260, *p* = 0.021), but not with SOFA scores in nervous system, cardio vascular system, coagulation, and renal system (all *p* > 0.05)). MALT1 expression (*p* = 0.010), Th1 cells (*p* = 0.010), Th17 cells (*p* = 0.038), and IL‐17A (*p* = 0.012), except for IFN‐γ (*p* = 0.102), elevated in sepsis deaths compared with sepsis survivors.

**Conclusion:**

PBMC MALT1 is highly expressed in sepsis patients with its overexpression associated with multiple organic dysfunctions, elevated Th17 cells, and increased mortality risk.

## Graphical abstract

In general, 78 sepsis patients and 40 HCs were enrolled. MALT1 expression was detected in PBMCs from all subjects by RT‐qPCR. Besides, Th1 and Th17 cells were measured in PBMCs from sepsis patients by flow cytometry; IL‐17A and IFN‐γ were determined in serum from sepsis patients by ELISA. MALT1 expression was higher in sepsis patients than HCs. MALT1 expression was positively correlated with Th17 cells and IL‐17A, but not with Th1 cells or IFN‐γ in sepsis patients. MALT1 expression was positively correlated with APACHE II score, CRP, SOFA respiratory system score, and SOFA liver score. MALT1 expression, Th1 cells, Th17 cells, and IL‐17A elevated in sepsis deaths compared with sepsis survivors.

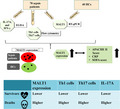

## 1 INTRODUCTION

Sepsis continues to be a global health problem with in‐hospital mortality ranging from 15.6% to 30%.^1^, ^2^, ^3^, ^4^, ^5^ Generally, sepsis is featured by severe, potentially fatal, multiple organic dysfunctions caused by dysregulated host response to infection; meanwhile, it is usually accompanied by inflammatory storm.^6^ Over the decades, though progressions have been made in the prevention and treatment of sepsis, its incidence still increases unexpectedly; meanwhile, its mortality rate remains unsatisfying.^5^, ^7^, ^8^, ^9^, ^10^ Considering the high mortality of sepsis, it would be valuable to find novel biomarkers for indicating disease severity and further realizing better management for sepsis patients.

Mucosa‐associated lymphoid tissue lymphoma translocation protein 1 (MALT1) is an intracellular signaling protein, with its coding gene located on chromosome 18 q21.^11^ Interestingly, previous studies illuminate that MALT1 is associated with dysregulated immune response to infection and multiple organic dysfunctions (including kidney injury, lung injury, and liver injury).^12^, ^13^, ^14^, ^15^, ^16^, ^17^ For instance, one study discloses that MALT1 regulates immune response in human primary T cells and macrophages via inactivating NEDD4‐binding protein 1 (N4BP1) and subsequently facilitates the viral reactivation of immunodeficiency virus (HIV)‐1.^12^ Beyond that, MALT1 overexpression could result in kidney injury via activating nuclear factor κB (NF‐κB) signaling.^15^ Additionally, it is illustrated that MALT1 enhances the differentiation of CD4^+^ T cells into Th1 and Th17 cells, meanwhile the differentiation of CD4^+^ T cells into Th1 and Th17, which are important in the pathology of sepsis.^18^, ^19^, ^20^, ^21^ However, the clinical relevance of MALT1 with sepsis, which is one of the most serious infection‐related diseases, still remains elusive.

Herein, the current study aimed to investigate the correlation of MALT1 with Th1 cells, Th17 cells, and prognosis in sepsis patients.

## 2 METHODS

### 2.1 Subjects

This study was a prospective cohort study. Between January 2019 and February 2021, this study serially enrolled 78 sepsis patients. The enrollment criteria for sepsis patients were as follows: (i) confirmed as sepsis in accordance with the sepsis‐3 criteria^22^; (ii) over 18 years old; (iii) hospitalized for sepsis treatment within 24h of symptom onset; and (iv) willing to provide peripheral blood (PB) samples. The exclusion conditions for sepsis patients were set as: (i) concomitant with autoimmune disease, hematological disease or solid tumor; and (ii) pregnant or gestational patient. Besides, from January 2019 to February 2021, the study also included 40 healthy subjects without abnormities in medical examinations as health controls (HCs), who were willing to provide PB samples for study use, and the exclusion conditions for sepsis patients were also suitable for health controls. The study protocol was approved by Ethics Committee.

### 2.2 Data collection

Demographics, disease characteristics, and biochemical indexes were collected for study analysis. Besides, Acute Physiology and Chronic Health Evaluation II (APACHE II) score and Sequential Organ Failure Assessment (SOFA) score were also obtained to evaluate the disease severity of sepsis patients. In addition, the 28‐day follow‐up information of sepsis patients was also collected, and mortality during follow‐up was calculated.

### 2.3 Sample preparation

For sepsis patients, PB samples were obtained after admission; then, peripheral blood mononuclear cells (PBMCs) were isolated by gradient density centrifugation using Ficoll PM400 (Cytiva), and serum samples were isolated by centrifuge at 3500 revolutions per minute for 10 mins. For HCs, PB samples were obtained after enrollment, and then PBMCs were separated.

### 2.4 Sample assessment

PBMCs of all sepsis patients as well as all HCs were used to determine MALT1 expression by reverse transcription quantitative polymerase chain reaction (RT‐qPCR). PBMCs of 51 sepsis patients were used to assess the proportion of T helper (Th)1 and Th17 cells (CD4^+^ T cells were considered as denominator in the calculation) by flow cytometry (FCM) using Human Cell Differentiation Kit (Bio‐Techne China Co. Ltd., China). Serum samples of all sepsis patients were used to evaluate the level of interferon gamma (IFN‐γ) and interleukin 17A (IL‐17A) by enzyme‐linked immunosorbent assay (ELISA) using commercial Human ELISA Kit (Bio‐Techne China Co. Ltd.). The experiments were carried out based on the manufacturer's instructions.

### 2.5 RT‐qPCR assay

MALT1 expression in PBMCs was detected with RT‐qPCR. In detail, the total RNA from PBMC was extracted using PureZOL RNA isolation reagent (Bio‐Rad); complementary DNA was synthesized by iScript™ cDNA Synthesis Kit (with random primer) (Bio‐Rad); qPCR was performed by SYBR^®^ Green Realtime PCR Master Mix (Toyobo). Besides, the current primers took reference from a previous study.^21^ Additionally, glyceraldehyde‐3‐phosphate dehydrogenase (GAPDH) was used as the internal reference; meanwhile, 2^−ΔΔCt^ method was applied to calculate the MALT1 relative expression.

### 2.6 Statistical analysis

Graphics and statistical analyses were completed using GraphPad Prism 7.02 (GraphPad Software Inc.) and SPSS 24.0 (IBM), respectively. Comparison of MALT1 expression between sepsis patients and HCs was analyzed using Wilcoxon rank‐sum test. The feasibility of using MALT1 expression as a detection index for the diagnosis of sepsis was determined by receiver operating characteristic (ROC) curves and the area under the ROC curve (AUC). Correlation of two variables was assessed using Spearman's rank correlation test, Kruskal–Wallis H rank‐sum test or Wilcoxon rank‐sum test. Among sepsis patients, differences of MALT1 expression, Th1 cells, IFN‐γ level, Th17 cells, and IL‐17A level between sepsis deaths and sepsis survivors were analyzed using Wilcoxon rank‐sum test, and the performance of clinical indexes in distinguishing sepsis survivors from sepsis deaths was evaluated using ROC curves and AUC. *p* value <0.05 was considered significant.

## 3 RESULTS

### 3.1 Clinical characteristics of sepsis patients

Among the enrolled 78 sepsis patients, the age was 56.6 ± 11.4 years with 49 (62.8%) males (Table 1). Additionally, the body mass index (BMI) was 23.3 ± 3.7 kg/m^2^. In terms of primary infection site, 28 (35.9%), 20 (25.6%), 16 (20.5%), and 14 (17.9%) patients had abdominal infection, respiratory infection, skin and soft tissue infection, as well as other infections, separately. In terms of general disease severity, APACHE II score and SOFA score were 12.6 ± 6.1 and 5.4 ± 2.5, respectively. More detailed information is mentioned in Table 1.

**TABLE 1:** Clinical characteristics

Items	Sepsis patients (*N* = 78)
Demographics	
Age (years), mean ± SD	56.6 ± 11.4
Male, *n* (%)	49 (62.8)
BMI (kg/m^2^), mean ± SD	23.3 ± 3.7
Smoke, *n* (%)	28 (35.9)
Drink, *n* (%)	30 (38.5)
History of disease, *n* (%)	
Hypertension	33 (42.3)
Hyperlipidemia	14 (17.9)
Diabetes	12 (15.4)
CKD	6 (7.7)
CCVD	21 (26.9)
Disease characteristics	
Primary infection site, *n* (%)	
Abdominal infection	28 (35.9)
Respiratory infection	20 (25.6)
Skin and soft tissue infection	16 (20.5)
Other infections	14 (17.9)
Primary organism, *n* (%)	
G‐ bacteria	47 (60.3)
G+ bacteria	24 (30.8)
Fungus	10 (12.8)
Others	9 (11.5)
Culture negative	11 (14.1)
APACHE II score, mean ± SD	12.6 ± 6.1
SOFA score, mean ± SD	5.4 ± 2.5
Respiratory system	1.3 ± 0.6
Nervous system	0.6 ± 0.6
Cardio vascular system	0.7 ± 0.6
Liver	1.0 ± 0.6
Coagulation	1.1 ± 0.6
Renal system	0.7 ± 0.7
Biochemical indexes	
CRP (mg/L), median (IQR)	89.3 (44.8–128.4)
Th1 cells (%), median (IQR)	16.8 (14.6–21.0)
IFN‐γ (pg/ml), median (IQR)	94.0 (72.2–124.0)
Th17 cells (%), median (IQR)	5.9 (5.0–6.3)
IL−17A (pg/ml), median (IQR)	149.1 (90.9–212.7)

Abbreviations: APACHE II, Acute Physiology and Chronic Health Evaluation II; BMI, body mass index; CCVD, cardiovascular and cerebrovascular diseases; CKD, chronic kidney disease; CRP, C‐reactive protein; G‐, Gram‐negative; G+, Gram‐positive; IFN‐γ, interferon gamma; IL‐17A, interleukin 17A; IQR, interquartile range; SD, standard deviation; SOFA, Sequential Organ Failure Assessment; Th1 cells, T helper 1 cells; Th17 cells, T helper 17 cells.

### 3.2 Comparison of PBMC MALT1 expression between sepsis patients and HCs

**FIGURE 1:**
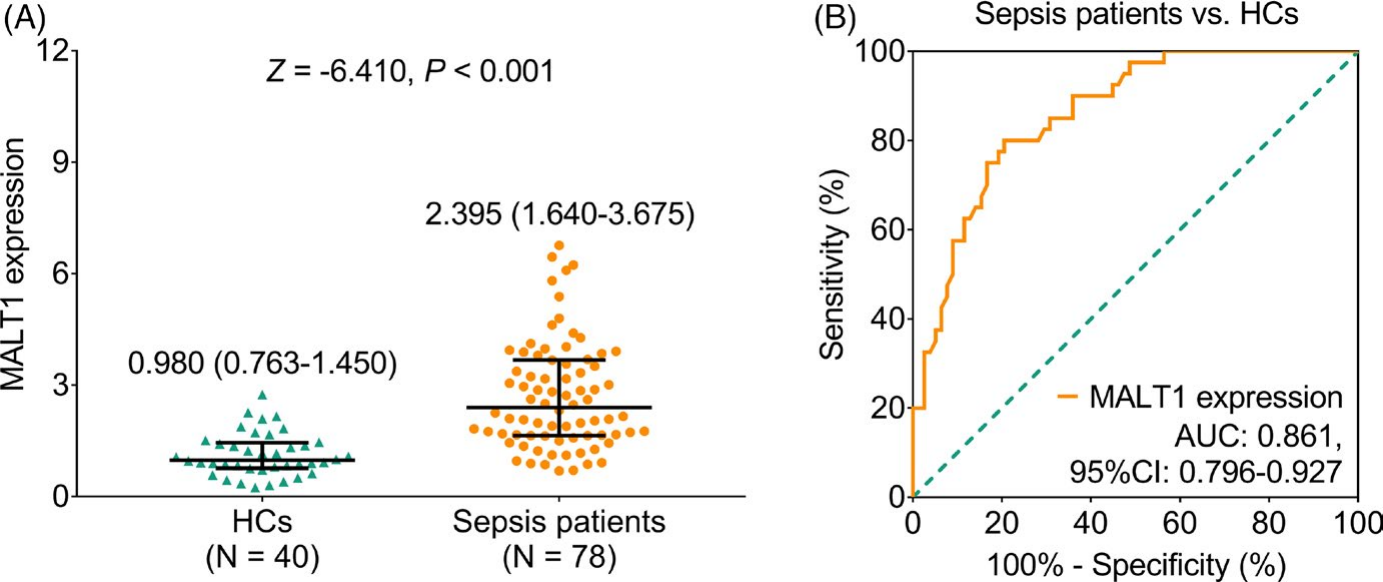
MALT1 expression was higher in sepsis patients compared with HCs. Comparison of MALT1 expression between sepsis patients and HCs (A); the ability of MALT1 expression to discriminate sepsis patients (B)

The median PBMC MALT1 expression in sepsis patients and HCs was 2.395 (interquartile range (IQR): 1.640–3.675) and 0.980 (IQR: 0.763–1.450), separately, which showed that PBMC MALT1 expression was higher in sepsis patients compared with HCs (*Z* = −6.410, *p* < 0.001, Figure 1A). Furthermore, the ROC curve presented that PBMC MALT1 exhibited a good ability in discriminating sepsis patients from HCs with area under curve (AUC) of 0.861 (95% confidence interval (CI): 0.796–0.927) (Figure 1B).

### 3.3 Association of PBMC MALT1 expression with Th1 cells, Th17 cells, and their secreted cytokines

**FIGURE 2:**
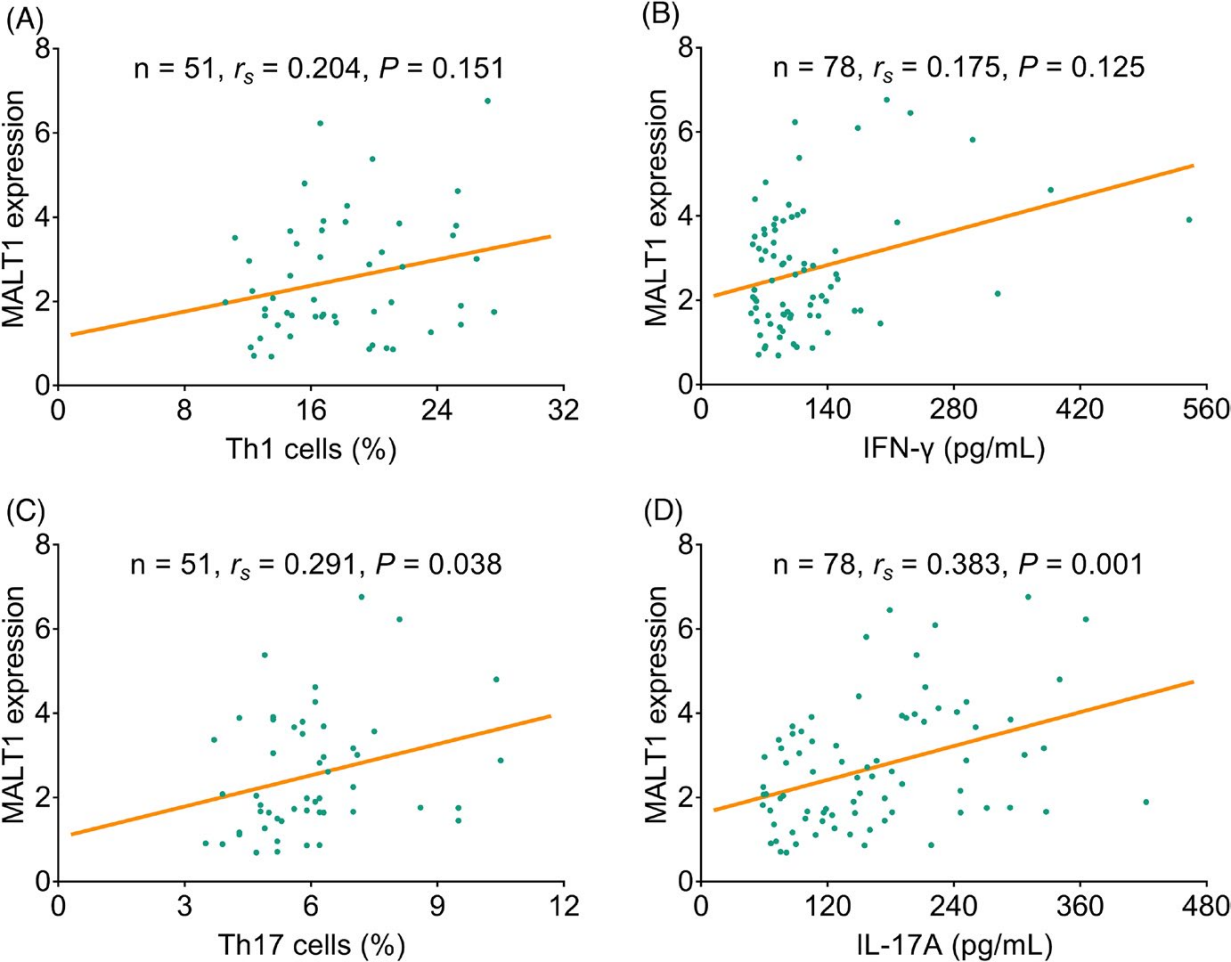
MALT1 expression positively linked with Th17 cells and IL‐17A in sepsis patients. Association of MALT1 expression with Th1 cells (A); IFN‐γ (B); Th17 cells (C); and IL‐17A (D)

PBMC MALT1 expression was positively correlated with Th17 cells (*r*_s_ = 0.291, *p* = 0.038) and IL‐17A level (*r*_s_ = 0.383, *p* = 0.001), but did not correlate with Th1 cells (*r*_s_ = 0.204, *p* = 0.151) or IFN‐γ level (*r*_s_ = 0.175, *p* = 0.125) (Figure 2A‐D).

### 3.4 Association of PBMC MALT1 expression with sepsis disease features

PBMC MALT1 expression was positively correlated with APACHE II score (*r*_s_ = 0.275, *p* = 0.015) and C‐reactive protein (CRP) (*r*_s_ = 0.257, *p* = 0.023) (Table 2). Besides, PBMC MALT1 expression was also positively correlated with SOFA score (*r*_s_ = 0.306, *p* = 0.006); in detail, PBMC MALT1 expression was positively linked with SOFA respiratory system score (*r*_s_ = 0.348, *p* = 0.002) and SOFA liver score (*r*_s_ = 0.260, *p* = 0.021) but not with other subscales (all *p* > 0.05). Beyond that, decreased PBMC MALT1 expression was correlated with the occurrence of G‐ bacteria‐primary infection (*Z* = −2.047, *p* = 0.041) but not correlated with primary infection sites or other primary organisms (all *p* > 0.05) (Table S1).

**TABLE 2:** Correlation of MALT1 with severity of disease in sepsis patients

Items	MALT1 expression	
	*r_s_*	*p* value
APACHE II score	0.275	0.015
SOFA score	0.306	0.006
Respiratory system	0.348	0.002
Nervous system	−0.015	0.896
Cardio vascular system	0.207	0.069
Liver	0.260	0.021
Coagulation	0.187	0.100
Renal system	0.210	0.065
CRP	0.257	0.023

Abbreviations: APACHE II, Acute Physiology and Chronic Health Evaluation II; CRP, C‐reactive protein; MALT1, mucosa‐associated lymphoid tissue lymphoma translocation protein 1; SOFA, Sequential Organ Failure Assessment.

### 3.5 Comparisons of PBMC MALT1 expression, Th1 cells, and Th17 cells between sepsis survivors and sepsis deaths

**FIGURE 3:**
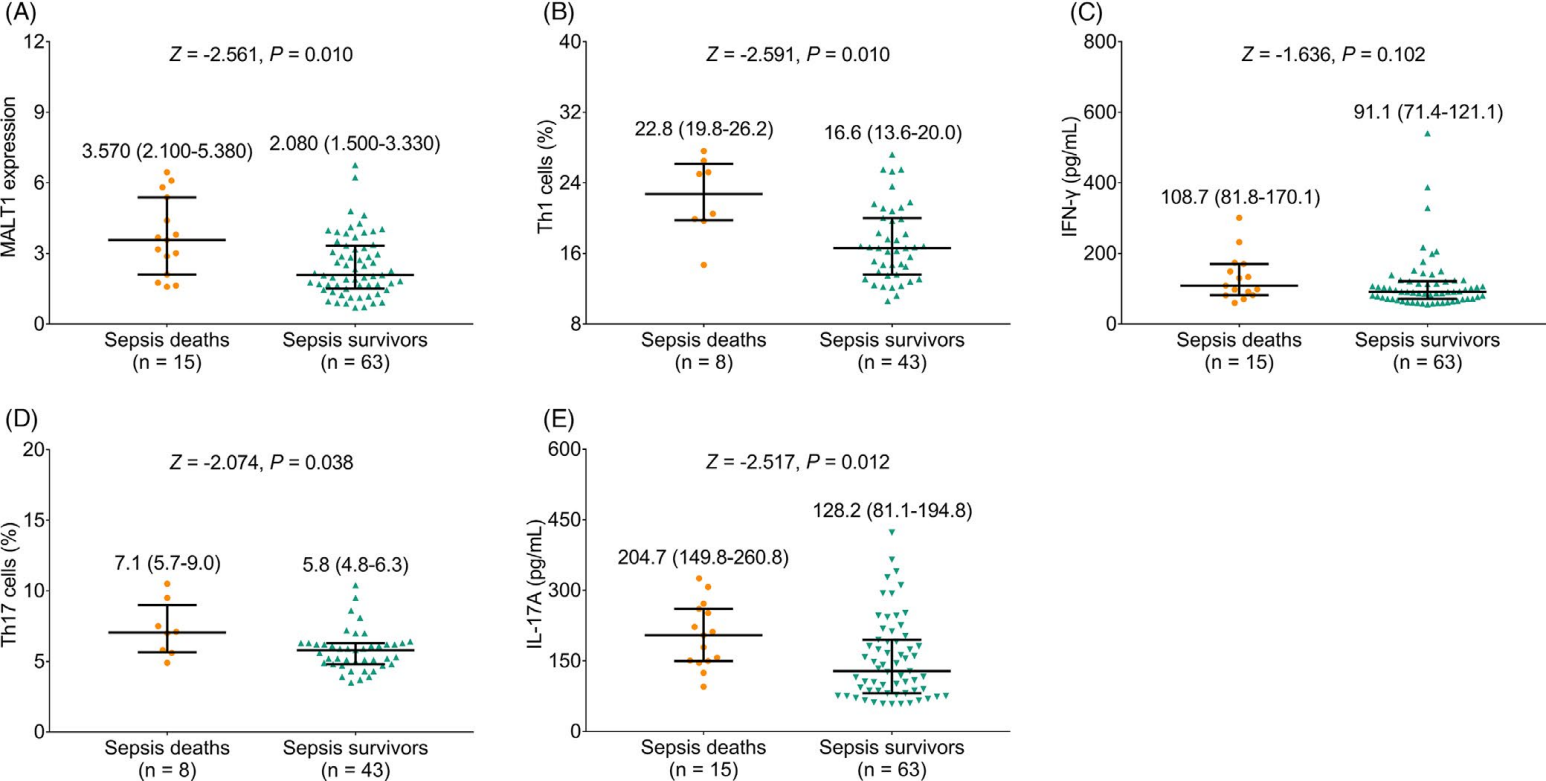
MALT1, Th1, and Th17 cells, as well as their secreted cytokines in sepsis deaths and sepsis survivors. Comparison of MALT1 expression (A); Th1 cells (B); IFN‐γ (C); Th17 cells (D); and IL‐17A (E) between sepsis deaths and sepsis survivors

PBMC MALT1 expression was elevated in sepsis deaths (3.570 (IQR: 2.100–5.380)) compared with sepsis survivors (2.080 (IQR: 1.500–3.330)) (*Z* = −2.561, *p* = 0.010, Figure 3A). Similarly, Th1 cells (*Z* = −2.591, *p* = 0.010), Th17 cells (*Z* = −2.074, *p* = 0.038), and IL‐17A level (*Z* = −2.517, *p* = 0.012), except for IFN‐γ (*Z* = −1.636, *p* = 0.102), were also elevated in sepsis deaths compared with sepsis survivors (Figure 3B‐E).

**FIGURE 4:**
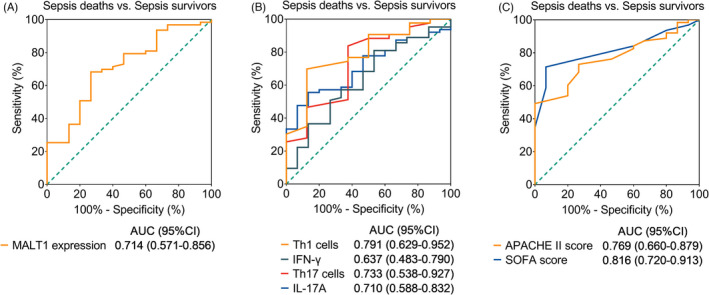
Indicators discriminating sepsis deaths from sepsis survivors. Discriminating value of MALT1 expression (A); Th1 cells, IFN‐γ, Th17 cells, and IL‐17A (B); APACHE II score and SOFA score (C) in differentiating sepsis deaths and sepsis survivors

Additionally, in terms of these indexes in discriminating sepsis deaths from sepsis survivors, PBMC MALT1 (Figure 4A) disclosed a similar value as Th1 cells, IFN‐γ, Th17 cells, and IL‐17A (Figure 4B) did, while these values were numerically inferior to APACHE II score and SOFA score (Figure 4C).

## 4 DISCUSSION

Recently, abnormally elevated MALT1 has been studied in a series of infection‐associated diseases such as acquired immune deficiency syndrome (AIDS), virulent rabies, and colitis,^12^, ^13^, ^14^ whereas its dysregulation in sepsis needs further exploration. In the current study, it was shown that PBMC MALT1 expression was higher in sepsis patients compared with HCs; meanwhile, MALT1 overexpression was linked with high sepsis risk. It could be possibly explained that: MALT1 overexpression is usually linked with strengthened infectivity of pathogenic microorganism in several infection‐associated diseases; meanwhile, sepsis is featured by systemic infection, thereby MALT1 overexpression is associated with higher sepsis risk.^23^, ^24^

Several previous studies disclose that MALT1 leads to T cell activation and differentiation into Th1 and Th17 cells in a series of infection‐associated diseases including colitis and encephalitis.^21^, ^25^, ^26^ In the current study, PBMC MALT1 expression was positively associated with Th17 cells, IL‐17A level in sepsis patients, but not with Th1 cells or IFN‐γ level. A possible explanation could be that: MALT1 facilitates the differentiation of CD4^+^ T cells into Th17 cells via activating NF‐κB signaling; therefore, MALT1 overexpression is linked with elevated Th17 cells and its secreted IL‐17A level.^25^, ^27^

Currently, SOFA scoring system is the main index for evaluating multiple organic dysfunctions in sepsis.^28^ However, previous studies never evaluate the correlation of MALT1 expression with this index in sepsis. Then, the present study disclosed that PBMC MALT1 expression was positively linked with general SOFA score, SOFA respiratory system score, and SOFA liver score in sepsis patients. 1) MALT1 might contribute to systemic inflammatory responses attributable to the infection in sepsis, which would probably lead to a significant lung injury; thus, MALT1 overexpression is correlated with unfavorable SOFA respiratory system score in sepsis patients; besides, MALT1 recruitment leads to caspase 1 activation and pyroptotic death of invariant natural killer T cells, which correspondingly lead to liver injury; thus, MALT1 overexpression is correlated with unfavorable SOFA respiratory system score and SOFA liver score in sepsis patients.^15^, ^16^, ^17^ 2) MALT1 expression is positively linked with the recruitment of Th17 cells and IL‐17A, which indicates sepsis severity.^29^, ^30^, ^31^, ^32^ Correspondingly, MALT1 overexpression might be correlated with sepsis severity in sepsis patients.

In the past decades, the mortality rate of sepsis still remains unsatisfying.^7^, ^8^, ^9^, ^10^ Hence, it is of great importance to investigate potential biomarkers to predict mortality risk, to further optimize the outcome of sepsis. The current study disclosed that the upregulation of MALT1, Th1 cells, Th17 cells, and IL‐17A was correlated with higher mortality risk in sepsis patients. Interestingly, discriminating value of MALT1 is similar to that of Th1 cells, IFN‐γ, Th17 cells, and IL‐17A; meanwhile, the discriminating value of MALT1 is somehow inferior to that of APACHE II score and SOFA score, which implies that MALT1 could serve as an assistant prognostic biomarker for sepsis. Possible explanations could be that: (1) MALT1 enhances the differentiation of Th1 cells and Th17 cells as well as their secreted cytokines, whose abnormal expressions are linked with aggravated sepsis severity and thereby be linked with elevated mortality risk.^21^, ^25^, ^26^, ^27^ Thus, elevated MALT1, together with Th1 cells, Th17 cells, and IL‐17A, is correlated with higher mortality risk in sepsis patients. (2) MALT1 dysregulation would lead to immunity error characterized by recurrent bacterial, viral, and fungal infections, periodontal disease, enteropathy, dermatitis, and failure to thrive. Subsequently, MALT1 is linked with mortality risk in sepsis patients.^33^ (3) The ability of MALT1 in estimating mortality risk is not as high as the APACHE II score and SOFA score. This phenomenon is inevitable, due to that the APACHE II is an accepted standard for evaluating the health status of sepsis patients, which would have a high ability in predicting the mortality risk of sepsis patients.

Additionally, MALT1 seems more correlated with Th17 but less correlated with Th1. Possible explanations for this could be that (1) MALT1 mainly facilitates the differentiation of regulatory T cells into Th17 cells instead of Th1 cells; thus, MALT1 seems more correlated with Th17 cells but less correlated with Th1 cells. (2) We merely enrolled 78 sepsis patients; thus, the statistical power of the correlation between MAlT1 and Th1 cells is somehow insufficient; hence, MALT1 seems more correlated with Th17 cells but less correlated with Th1 cells.

Some limitations still existed in our research, for instance: (1) the sample size of the current study was relatively small, which would probably result in a less strong statistical power in analysis. (2) The current study was a single‐center research; thus, a selection bias might exist. (3) MALT1 expression should be detected at multi‐time point to assess its value in monitoring the disease progression for sepsis patients in the forthcoming study. (4) The sepsis patients enrolled had a mean age of 56.6 ± 11.4 years; thus, our results might not be applicable for younger sepsis patients. (5) The current study did not enroll neonatal sepsis patients, and this area needed to be further explored in future.

Collectively, PBMC MALT1 is highly expressed in sepsis patients with its overexpression associated with multiple organic dysfunctions, elevated Th17 cells, and increased mortality risk, which implies that it could forecast the prognosis of sepsis patients.

## CONFLICTS OF INTEREST

The authors declare that they have no conflicts of interest.

## Supporting information

Table S1

## Data Availability

Data sharing is not applicable to this article as no datasets were generated or analyzed during the current study.
