# Supplementary material for: Aberrant blood MALT1 and its relevance with multiple organic dysfunctions, T helper cells, inflammation, and mortality risk of sepsis patients
Source: J Clin Lab Anal. 2022 Mar 9;36(4):e24331. doi: 10.1002/jcla.24331 (PMC8993658; doi:10.1002/jcla.24331)
Supplement: Supplementary file 1 — Table S1 [file JCLA-36-e24331-s001.docx]

**Supplementary Table 1.** Correlation of MALT1 with primary infection site and primary organism in sepsis patients.

| Items | MALT1 expression | | |
| --- | --- | --- | --- |
|  | Median (IQR) | Statistic (*H* /*Z*) | *P* value |
| **Primary infection site** |  | 3.195 | 0.363 |
| Abdominal infection | 2.275 (1.643-3.425) |  |  |
| Respiratory infection | 2.240 (1.663-3.905) |  |  |
| Skin and soft tissue infection | 2.115 (1.180-3.185) |  |  |
| Other infections | 2.895 (1.743-5.123) |  |  |
| **Primary organism** |  |  |  |
| G- bacteria |  | -2.047 | 0.041 |
| Negative | 3.050 (1.670-3.940) |  |  |
| Positive | 2.040 (1.500-3.330) |  |  |
| G+ bacteria |  | -0.227 | 0.820 |
| Negative | 2.395 (1.638-3.813) |  |  |
| Positive | 2.410 (1.643-3.520) |  |  |
| Fungus |  | -1.771 | 0.077 |
| No | 2.205 (1.633-3.475) |  |  |
| Yes | 3.555 (1.735-6.125) |  |  |
| Others |  | -0.055 | 0.956 |
| No | 2.470 (1.635-3.745) |  |  |
| Yes | 2.080 (1.825-3.210) |  |  |
| Culture Negative |  | -1.902 | 0.057 |
| No | 2.100 (1.640-3.370) |  |  |
| Yes | 3.800 (2.160-5.380) |  |  |

MALT1, mucosa-associated lymphoid tissue lymphoma translocation protein 1; IQR, interquartile range; G-, Gram-negative; G+, Gram-positive.
